# Differences of Clinical Characteristics and Drug Prescriptions between Men and Women with COPD in China

**DOI:** 10.3390/toxics11020102

**Published:** 2023-01-21

**Authors:** Yuqin Zeng, Martijn A Spruit, Qichen Deng, Frits M. E. Franssen, Ping Chen

**Affiliations:** 1 Department of Pulmonary and Critical Care Medicine, The Second Xiangya Hospital, Central South University, Changsha 410011, China; 2 Research Unit of Respiratory Disease, Central South University, Changsha 410011, China; 3 Hunan Centre for Evidence-Based Medicine, Changsha 410011, China; 4 Department of Research & Development, CIRO, 6085 NM Horn, The Netherlands; 5 Department of Respiratory Medicine, Maastricht University Medical Centre (MUMC+), 6229 HX Maastricht, The Netherlands; 6 NUTRIM School of Nutrition and Translational Research in Metabolism, 6229 ER Maastricht, The Netherlands

**Keywords:** COPD, sex difference, symptom, prescription

## Abstract

Background: Sex differences in symptoms exist in patients with COPD. Our aim is to measure the differences between men and women with COPD, focusing on risk factors, symptoms, quality of life and drug prescriptions. Methods: In this cross-sectional observational study, patients with COPD were collected in China; demographic characteristics, smoking history, occupational exposure, biomass exposure, lung function, dyspnea, quality of life, and prescriptions for inhaled medications were collected. The nearest neighbor algorithm was used to match female and male patients (ratio 2:1) on age, body mass index, and lung function. Results: Compared with 1462 men, the 731 women generally had lower educational levels and were married less (both *p* < 0.001). A total of 576 (90.0%) women did not smoke cigarettes. More men were exposed to occupational dust (539 (36.9%) vs. 84 (11.5%), *p* = 0.013), while more women were exposed to biomass smoke (330 (45.1%) vs. 392 (26.8%), *p* = 0.004). Except for phlegm and chest tightness, women had more complaints than men for cough, breathlessness, activities, confidence, sleep and energy (*p* < 0.05). In addition, more women were prescribed triple therapy than men (236 (36.3%) vs. 388 (31.0%), *p* = 0.020). Conclusions: There are obvious discrepancies in the quality of life and use of inhaled medications between male and female patients with COPD.

## 1. Introduction

Chronic obstructive pulmonary disease (COPD) is a common chronic respiratory disease characterized by progressive and mostly irreversible airflow limitation caused by exposure to tobacco smoke, occupational or environmental toxins, and/or biomass combustion products [1]. Based on some large-scale epidemiological studies, the number of COPD cases was 384 million in 2010, with a global prevalence of 11.7% (8.4–15.0%) in adults aged 30 years or older [2]. In China, approximately 100 million patients aged 20 years or older were living with diagnosed COPD in 2015, with an overall prevalence of 8.6%; among the general population aged 40 years or older, the prevalence of COPD was 13.7% [3].

Among smokers, the prevalence of COPD was higher in men (17.1% versus 13.2%) than women, while among never smokers the distributions of COPD by age were similar between men and women (5.2% vs. 6.2%) [4]. Among the non-smoker COPD population, more than 80% were women who may spend more time indoors for cooking, and thus are more exposed to biomass fuel combustion products than men [5]. More men were exposed to occupational risk factors than women in patients with COPD in China [6]. Sex differences of expressions in COPD existed, with women experiencing more exacerbations, having poorer health status, and different comorbidities [7,8]. A study that matched females to males on forced expiratory volume in one second (FEV_1_% predicted) found that females were younger, less likely to be smokers, and more breathless [9]. However, their sample size was small and they did not consider other risk factors.

A deeper understanding of the differences in the clinical expression of COPD between women and men is needed, as sex-related differences could facilitate more personalized disease management [10]. Few studies have investigated sex-related bias in medication prescribing. Dales et al. [11] found that females with moderate COPD were twice as likely as males to be prescribed medications; there was no difference in medication use in men and women with severe COPD. However, they did not show the detailed prescriptions for the patients in men and women.

In this cross-sectional observational study, we systematically evaluated the differences between male and female patients for demographics, symptoms, quality of life and drug prescriptions for COPD. At the same time, the relative factors of complaints were calculated by gender.

## 2. Materials and Methods

### 2.1. Study Design and Data Source

This was a cross-sectional observational study using the database created by the Second Xiangya Hospital of Central South University (http://120.77.177.175:9007/a/login, accessed on 17 February 2022). We collected data from December 2016 to December 2021 in 13 hospitals (11 hospitals in Hunan Province and 2 in Guangxi Province) in China. The institutional review board from the Second Xiangya Hospital of Central South University supported the study (2016076). We identified that this research was conducted in accordance with the Declaration of Helsinki and has been registered in the Chinese Clinical Trial Registry (ChiCTR-POC-17010431). All participants provided written informed consent. Two technical assistants cross-checked all of the data to eliminate typing omission and errors.

### 2.2. Participants

Eligible participants were all patients diagnosed with COPD according to the medical records, at least 35 years of age, and visited the pneumological outpatient clinic. Patients were excluded if they had a diagnosis of asthma, bronchiectasis, or lung cancer during the study period. Basic variables of all subjects were collected, including demographics, diagnosis, exacerbation history, lung function test, COPD Assessment Test (CAT) score [12], modified Medical Research Council (mMRC) [13] score and current prescriptions. Prescriptions were recorded including short-acting bronchodilators (SABDs): short-acting β_2_-agonists (SABAs) and short-acting muscarinic agonists (SAMAs); long-acting bronchodilators (LABDs): long-acting β_2_-agonists (LABAs) and long-acting muscarinic agonists (LAMAs); and other inhalations: inhaled corticosteroid (ICS); dual therapy including LAMA combined with LABA (LAMA/LABA) and LABA combined with ICS; and triple therapy (LAMA + LABA + ICS) [1].

Since the prevalence of COPD is higher in men than women (nearly two times) in China [3], the nearest neighbor algorithm was used to match male to female as a ratio of 2:1 on age, body mass index (BMI), and FEV_1_% predicted.

### 2.3. Exposure History

Smoking history was recorded in this study. “Never smokers” were those who had never smoked a cigarette or smoked less than 100 cigarettes in their life; “current smokers” were those who had smoked ≥100 cigarettes in their life and currently smoked cigarettes; and “former smokers” were those who had smoked ≥100 cigarettes in their life but did not currently smoke and had quit smoking longer than 6 months [14].

Occupational exposures were calculated directly from self-reported exposure, which was based on definite answers to the item “Have you ever been exposed at your workplace to vapor, gas, dust, or fumes?” [15]. If the answer was “yes”, the duration and category of occupational exposure would be recorded. Cumulative exposure was also assessed for each exposure type by multiplying exposure hours and the number of years worked in the specific job (hour-years) [16].

Biomass exposure was estimated directly from the self-reported exposure which was based on affirmative answers to the item “Have you ever used biomass fuels (wood, grass, charcoal, crop residues and animal wastes) for cooking or heating or other things?” If the answer was “yes”, the duration and category of biomass exposure would be collected. Cumulative exposure was also expressed as multiplying exposure hours and the number of years (hour-years) [17,18].

### 2.4. Exacerbations

Exacerbations were defined as an acute worsening of respiratory symptoms that results in additional therapy. Exacerbations can be classified into three levels: mild (treated with short-acting bronchodilators only, SABDs), moderate (treated with SABDs plus antibiotics and/or oral corticosteroids), and severe (patient requires hospitalization or visits the emergency room) [1,19]. In our research, patient estimates of moderate and severe exacerbation numbers in the past year before they visited were recorded [20].

### 2.5. Statistical Analysis

The nearest neighbor algorithm was used to match the male to female as a ratio of 2:1 on age, BMI, and FEV_1_% predicted. Data analyses were performed using PASW version 18.0 software (PASW, Inc., Chicago, IL, USA). The baseline characteristics of the subjects were described using mean ± standard deviation (SD) or median (interquartile range, IQR). Results were stratified by sex and differences were calculated either with an independent t-test or chi-squared or nonparametric Mann–Whitney tests. Mean substitution was used to replace the missing value to avoid data bias. Spearman’s rank correlation coefficient was used to measure the correlations of CAT. We considered a *p*-value less than 5% as statistically significant.

### 2.6. Patient and Public Involvement

Patients and/or the public did not take part in the development, conduct, reporting or dissemination of this study.

## 3. Results

### 3.1. Baseline and Clinical Characteristics

After matching, 1462 men and 731 women were enrolled in the current analyses (Table 1). Compared with men, women had lower educational levels and were less often married (*p* < 0.001, *p* < 0.001). Without the missing data, 576 (90.0%) of women did not smoke cigarettes, compared with 153 (10.8%) of men (*p* < 0.001). There were significant differences between men and women in occupational and biomass exposure. A total of 539 (36.9%) of men in COPD were exposed to occupational factors, compared to 84 (11.5%) of women (*p* < 0.001), while 330 (45.1%) of women in COPD were exposed to biomass, compared to 392 (26.8%) of men (*p* < 0.001). Moreover, the cumulative exposure time of occupation was longer in men than women (120 (60, 240) vs. 80 (50, 180) hour-years, *p* = 0.013). The cumulative exposure time of biomass was longer in women than men (70 (40, 120) vs. 60 (30, 100) hour-years, *p* = 0.004).

Female patients had better FEV_1_ to forced vital capacity (FEV_1_/FVC) than males (55.0 ±12.6 vs. 52.4 ± 12.5, *p* < 0.001), even though matching included FEV_1_% predicted. In addition, women had higher CAT scores and mMRC scores than men (16.5 ± 6.7 vs. 15.1 ± 6.8, *p* < 0.001; 2 (1, 3) vs. 2 (1, 2), *p* = 0.03). There was no significant difference in exacerbations between men and women (*p* = 0.174).

Women had more complaints than men about cough, breathlessness, activities, confidence, sleep and energy (Table 2, *p* < 0.05), except phlegm and chest tightness (*p* > 0.05). The age, exacerbations, and biomass exposure associated with CAT scores were weakly positive both in men and women (see Supplement Material, *p* < 0.05), contrary to BMI, educational level, and FEV_1_% predicted with a negative relationship (*p* < 0.05).

### 3.2. Drug Prescriptions

More men at a total of 138 (9.4%) did not use inhaled medications compared to 42 (5.7%) women (*p* = 0.003) without the missing data (73 (5.0%) and 39 (5.3%), respectively) (Table 3). More men had monotherapy, especially LAMA (517 (41.33%) vs. 229 (35.23%), *p* = 0.010). More women were prescribed triple therapy than males (236 (36.31%) vs. 388 (31.02%), *p* = 0.020).

## 4. Discussion

After matching women and men with COPD on age, BMI, and FEV_1_% predicted, we found that women had lower educational levels, were less frequently smokers, had less time of occupational exposure, a higher rate of widowhood, a higher FEV_1_/FVC ratio, more time of biomass exposure and more symptoms than men, especially in cough, dyspnea, activities, confidence, sleep and energy. Moreover, more women had been prescribed triple therapy.

Low education is an independent risk factor for COPD [3]. Nearly 50% of our sample had a primary school educational level or below, especially in women (59%). In the age of the study population (64 years old), there was a huge gender gap in educational attainment, especially in rural areas in China. Many factors, such as sex-related disparity in household expectations about education, gender blindness in setting up schools, and limited funds, led a lot of female students to drop out of their studies after elementary school [21,22]. There were also gender differences in cigarette smoking and occupational and biomass exposure. In 2010, an estimated prevalence ratio of 52.9% in men and 2.4% in women were current smokers in China [23], the same as the population of our study. In Sweden, the population attributable distribution for COPD from occupational exposure to particles was larger in men than women (10.6% vs. 6.1%) [24]. In our study, there were three times the number of men than women (36.9% vs. 11.5%) who were exposed to occupational factors. However, most women had been exposed to biomass fuel for cooking food or heating using wood, grass, and charcoal. Biomass exposure is a key risk factor for women in the development of COPD [25,26].

Several researchers have reported that women have more dyspnea and are less likely to report phlegm [27,28]. Our study found that women suffered from more cough and breathlessness. While there was no sex-related difference in phlegm and chest tightness. Inactivity in daily life is markedly common in patients with COPD [29]. More women with COPD were affected in terms of activity levels and have less energy in this study, which is consistent with Sonia’s study [30]. The reasons may be that muscle dysfunction, muscle wasting, and weakness are more marked in women than men [31,32], and most women have to change their functional performance, particularly for the heaviest chores [33]. Sleep disturbances are very common symptoms in patients with COPD [34]. More women complained that they had problems with sleep in our study, which may be attributed to fluctuating sex hormones, anxiety, or depression [35].

Many factors can be contributed to the serious symptoms and quality of life with COPD, such as lower lung function and higher age [36]. In our study, we found that older age, lower BMI, lower educational level, more acute exacerbations, longer time of biomass exposure and lower lung function were weakly association with a worse quality of life, no matter in men and women. A study of Lee SD [37] also demonstrated that a history of exacerbations in the preceding year is associated with CAT scores which can identify patients at further risk of exacerbations in the following six months. In addition, other studies have shown that biomass exposure has a negative impact on the quality of life in women patients with COPD [38].

Women are more likely than men to be prescribed respiratory medications [11]. Our finding of women’s higher likelihood to be prescribed triple therapy than men is consistent with a Swedish study on COPD [39]. Moreover, the majority of men had received the monotherapy of LAMA in our study. This phenomenon can be explained by the greater attention and increased actions by women with COPD who are more likely to apply for care [40]. Another explanation is that women with COPD present with more symptoms may have an impact on the doctor’s perception of using more combinations of inhalations according to the guidelines [41].

Finally, our study has some limitations. First, exposures to risk factors were self-reported by the patients which may cause recall bias. We arranged the same research assistant to record the data but we still cannot avoid this bias. Second, we only used the CAT and mMRC to assess the symptoms and quality of life for patients with COPD. As we all know, the CAT is not as complicated as St. George’s Respiratory Questionnaire, but it is similar to it [42]. CAT is simple and more practical to be implemented in clinics [43]. Third, we just collected the prescriptions of inhaled bronchodilators for patients with COPD because they are the essential treatments for COPD. In addition, we did not collect the data of blood eosinophil because at the beginning of our study the blood test for eosinophils was not common at outpatient clinics in China.

In conclusion, this study indicates that independent of age, BMI, and impairment of lung function, there are still important gender differences in the impact of COPD. Women were lower educated, less frequently smokers, less exposed to occupational factors, but underwent higher biomass exposure, experienced more symptoms, and received more triple therapy. Our findings highlight that clinical characteristics with regard to gender influence guideline implementation for patients with COPD. Further studies need to explore the implications of the sex-related differences in the outcomes of therapy.

## Figures and Tables

**Table 1:** Baseline and clinical characteristics of COPD patients.

Baseline Clinical Characteristics	Men *n* = 1462	Missing Data	Women *n* = 731	Missing Data	*p*-Value
Age (year), mean ± SD	64.4 ± 9.4	0 (0)	64.45 ± 9.7	0 (0)	-
Male, *n* (%)	-	-	-	-	
BMI (kg/m^2^), mean ± SD	22.8 ± 3.5	0 (0)	22.9 ± 4.0	0 (0)	-
Educational level, *n* (%)		51 (3.5)		40 (5.5)	<0.001
Primary school or below	611 (43.3)		410 (59.3)		
Middle school	523 (37.1)		186 (26.9)		
High school	201 (14.2)		73 (10.6)		
College or above	76 (5.4)		22 (3.2)		
Marital status, *n* (%)		43 (2.9)		36 (4.9)	<0.001
Unmarried	12 (.8)		4 (.6)		
Married	1346 (94.9)		611 (87.9)		
Divorced	5 (.4)		5 (.7)		
Widowhood	56 (3.9)		75 (10.8)		
Smoking history, *n* (%)		49 (3.4)		91 (12.4)	<0.001
Never smoker	153 (10.8)		576 (90.0)		
Former smoker	595 (42.1)		29 (4.5)		
Current smoker	665 (47.1)		35 (5.5)		
Occupational exposure (*n*, %)	539 (36.9)	114 (7.8)	84 (11.5)	59 (8.1)	<0.001
Exposure time (h-y)	120 (60, 240)		80 (50, 180)		0.013
Biomass exposure (*n*, %)	392 (26.8)	108 (7.4)	330 (45.1)	54 (7.4)	<0.001
Exposure time (h-y)	60 (30, 100)		70 (40, 120)		0.004
Lung function, mean ± SD		0 (0)		0 (0)	
FEV_1_% predicted	57.0 ± 21.8		57.1 ± 22.0		-
FEV_1_/FVC%	52.4 ± 12.5		55.0 ±12.6		<0.001
CAT, mean ± SD	15.1 ± 6.8	46 (3.1)	16.5 ±6.7	42 (5.7)	<0.001
<10 points	306 (21.6)		104 (15.1)		
10–20 points	760 (53.7)		363 (52.7)		
≥20 points	350 (24.7)		222 (32.2)		
mMRC dyspnea grade, median (IQR)	2 (1–2)	46 (3.1)	2 (1–3)	42 (5.7)	0.03
0 or 1	505 (35.7)		234 (34.0)		
≥2	911 (64.3)		455 (66.0)		
Exacerbation history in last one year, median (IQR)	1 (0–2)	80 (5.5)	1 (0–2)	75 (10.3)	0.174

Note: BMI, body mass index; FEV_1_, forced expiratory volume in one second; FEV_1_/FVC%, FEV_1_ to the forced vital capacity; CAT, COPD Assessment Test; mMRC, modified Medical Research Council; SD, standard deviation; IQR, interquartile range. T-test was used for numerical variables; non-parametric or chi-square test for categorical variables, *p* <0.05.

**Table 2:** Comparisons between men and women in CAT.

CAT, Median (IQR)	Men *n* = 1416	Women *n* = 689	*p*-Value
CAT1-Cough	2 (1–3)	2 (1–3)	0.041
CAT2-Phlegm	2 (1–3)	2 (1–3)	0.141
CAT3-Chest tightness	2 (1–3)	2 (1–3)	0.291
CAT4-Breathlessness	2 (1–3)	2 (1–4)	<0.001
CAT5-Activities	2 (1–3)	3 (2–3)	0.038
CAT6-Confidence	0 (0–2)	1 (0–2)	<0.001
CAT7-Sleep	1 (0–3)	2 (1–3)	<0.001
CAT8-Energy	2 (1–3)	3 (2–3)	0.038

Note: CAT, COPD Assessment Test; IQR, interquartile range. Non-parametric was used.

**Table 3:** Prescriptions in men and women with COPD.

Therapies, Only Used, *n* (%)	Men *n* = 1251	Women *n* = 650	*p*-Value
Mono therapy	517 (41.33)	229 (35.23)	0.010
SABA	21	1	
SAMA	5	1	
LABA	0	0	
LAMA	490	226	
ICS	1	1	
Dual therapy	273 (21.82)	153 (23.54)	0.395
LABA/ICS	162	110	
LAMA/LABA	109	43	
LAMA + ICS	2	0	
Triple therapy	388 (31.02)	236 (36.31)	0.020
LABA/LAMA/ICS	50	17	
LABA/LAMA + ICS		1	
LAMA + LABA/ICS	338	218	
Other therapies	73 (5.84)	32 (4.92)	0.409

Note: SABA, short-acting β_2_-agonist; SAMA, short-acting muscarinic agonist; LABA, long-acting β_2_-agonist; LAMA, long-acting muscarinic agonist; ICS, inhaled corticosteroid. Chi-square was used for the comparisons, *p* < 0.05.

## Data Availability

The datasets generated during and/or analyzed during the current study are available in the Department of Pulmonary and Critical Care Medicine, the Second Xiangya Hospital repository http://218.4.234.74:9007/a/login, accessed on 17 February 2022. The data that support the findings of this study are available upon reasonable request from the authors Yuqin Zeng or Ping Chen within three years.

## Supplementary Materials

The following supporting information can be downloaded at: https://www.mdpi.com/article/10.3390/toxics11020102/s1, Table S1: Correlations of CAT in men and women.
