# Supplementary material for: Differences of Clinical Characteristics and Drug Prescriptions between Men and Women with COPD in China
Source: Toxics. 2023 Jan 21;11(2):102. doi: 10.3390/toxics11020102 (PMC9967702; doi:10.3390/toxics11020102)
Supplement: Supplementary file 1 [file toxics-11-00102-s001.zip › toxics-2134302-supplementary.pdf]

Table S1. Correlations of CAT in men and women.

	CAT1- Cough		CAT2- Phlegm		CAT3- Chest tightness		CAT4- Breathlessness		CAT5- Activities		CAT6- Confidence		CAT7- Sleep		CAT8- Energy		Sum of CAT	
	**Men**	**Women**	**Men**	**Women**	**Men**	**Women**	**Men**	**Women**	**Men**	**Women**	**Men**	**Women**	**Men**	**Women**	**Men**	**Women**	**Men**	**Women**
**Age**	0.060*	0.042	0.055*	0.023	0.022	0.029	0.176*	0.190*	0.203*	0.160*	0.139*	0.218*	0.005	0.050	0.203*	0.160*	0.191*	0.178*
**BMI**	−0.124*	−0.003	−0.117*	−0.036	−0.062*	−0.032	−0.111*	−0.041	−0.128*	−0.084*	−0.108*	−0.044	−0.050	−0.033	−0.128*	−0.084*	−0.168*	−0.081*
**Educational level**	−0.056*	−0.045	−0.022	−0.002	−0.009	−0.036	−0.122*	−0.179*	−0.139*	−0.145*	−0.076*	−0.087*	−0.060*	−0.020	−0.139*	−0.145*	−0.134*	−0.149*
**Marital status**	0.002	−0.075	−0.019	−0.120*	−0.013	0.004	0.028	0.035	0.024	0.074	−0.013	−0.023	−0.001	0.040	0.024	0.074	0.010	0.025
**AE**	0.134*	0.140*	0.133*	0.086*	0.151*	0.069*	0.206*	0.151*	0.212*	0.282*	0.166*	0.125*	0.159*	0.135*	0.212*	0.282*	0.282*	0.274*
**Cigarette smoking**	−0.011	−0.010	0.018	−0.010	−0.019	−0.006	0.041	0.071	0.134*	0.052	−0.032	0.095*	−0.065*	0.021	0.134*	0.052	0.059*	0.065
**Occupational exposure**	0.083	−0.040	0.039	−0.026	−0.013	0.046	0.100*	−0.027	0.109*	−0.114	−0.015	−0.003	0.053	−0.077	0.109*	−0.114	0.101*	−0.095
**Biomass exposure**	0.063	0.132*	0.014	0.087	0.147*	0.110*	0.147*	0.149*	0.074	0.106*	−0.056	0.041	−0.051	0.047	0.074	0.106*	0.118*	0.150*
**FEV1%pre**	−0.152	−0.079	−0.194*	−0.161*	−0.144*	−0.161*	−0.340*	−0.339*	−0.304*	0.352*	−0.221*	−−0.243*	−0.088*	−0.049	−0.304*	−352*	−0.360*	−0.367*

Note: BMI, body mass index; AE: acute exacerbation; FEV_1%pre: forced expiratory volume in one second percent predicted. Spearman's rank correlation coefficient was used, **P*<0.05
